# Gender differences in trauma, shock and sepsis

**DOI:** 10.1186/s40779-018-0182-5

**Published:** 2018-10-26

**Authors:** Florian Bösch, Martin K. Angele, Irshad H. Chaudry

**Affiliations:** 1 Department of General, Visceral, and Transplant Surgery, Ludwig Maximilians-University Munich, 81377 Munich, Germany; 2 Center for Surgical Research and Department of Surgery, University of Alabama at Birmingham, Birmingham, AL 35294 USA

**Keywords:** Trauma-hemorrhage, Cardiopulmonary bypass, Gender morphism, Hormonal milieu, Estrogens

## Abstract

Despite efforts in prevention and intensive care, trauma and subsequent sepsis are still associated with a high mortality rate. Traumatic injury remains the main cause of death in people younger than 45 years and is thus a source of immense social and economic burden. In recent years, the knowledge concerning gender medicine has continuously increased. A number of studies have reported gender dimorphism in terms of response to trauma, shock and sepsis. However, the advantageous outcome following trauma-hemorrhage in females is not due only to sex. Rather, it is due to the prevailing hormonal milieu of the victim. In this respect, various experimental and clinical studies have demonstrated beneficial effects of estrogen for the central nervous system, the cardiopulmonary system, the liver, the kidneys, the immune system, and for the overall survival of the host. Nonetheless, there remains a gap between the bench and the bedside. This is most likely because clinical studies have not accounted for the estrus cycle. This review attempts to provide an overview of the current level of knowledge and highlights the most important organ systems responding to trauma, shock and sepsis. There continues to be a need for clinical studies on the prevailing hormonal milieu following trauma, shock and sepsis.

## Background

Despite efforts in resuscitation measures and intensive care, acute trauma and the resulting shock and subsequent sepsis remain associated with a high mortality [1]. A great deal of work has also been done in the prevention of traumatic injury. Nevertheless, traumatic injury is the major cause of death in people younger than 45 years of age and thus remains a major public issue [2–4]. Traumatic brain injury (TBI) accounts for 25% of long-term disabilities in individuals younger than 35 years of age. With an estimated annual incidence of 1.7 million individuals in the United States and a cost of $76.1 billion, TBI is a major social and economic burden [3, 5, 6].

Severe blood loss, often linked to traumatic injury, is associated with a high morbidity and mortality. The US Armed Forces reported 4,596 battlefield deaths from 2001 to 2011. Of these casualties, only 13% reached medical facilities prior to death. The authors classified 24% of the premedical facility deaths as potentially survivable, of which 90% were due to severe hemorrhage [7, 8]. Hemorrhagic shock and subsequent hypoperfusion to the body lead to hypoxia and eventual death. Therefore, controlling blood loss and administering resuscitative fluids are standard recommendations for the treatment of major blood loss [9]. In remote, distant military situations, management of hemorrhagic shock is challenging since large fluid volumes cannot be routinely supplied. Therefore, the US Department of Defense is supporting research to improve medical treatment on the battlefield [10]. In this respect, experimental animal studies have demonstrated that a single, small-volume infusion of ethinyl estradiol-3-sulfate (EES) has beneficial effects following trauma-hemorrhage, even in the absence of fluid resuscitation [11, 12].

Survivors of severe blood loss concomitant with trauma have a high risk of developing subsequent sepsis and multiple organ failure. Regardless of outstanding advances in the understanding and treatment of sepsis, the mortality rate remains at 30% [13, 14]. In the last decades, numerous studies have demonstrated gender dimorphism in response to trauma and sepsis with respect to immunological, cardiovascular and pathophysiological mechanisms [15–20]. Several studies have reported that women are less susceptible to posttraumatic infections and multiple organ failure [21–24]. A large body of evidence from animal studies definitively supports these findings [11, 12, 25–27]. The more favorable outcome in female patients following trauma and blood loss is mediated via sex hormones and in particular, the binding of estrogen to the estrogen receptors [10, 26, 28, 29].

Given the previously demonstrated gender differences following trauma and shock in experimental (*in vitro* as well as *in vivo*) and clinical studies, it is essential that future studies take gender into account. Since May of 2014, the National Institutes of Health (NIH) accordingly requires information about the composition of cells and animal gender in preclinical studies.

In addition, there is an apparent genetic disparity since females carry two inherently polymorphic X chromosomes, while males have only one polymorphic X chromosome passed from the mother [30–33].

## Gender dimorphism in trauma, shock and sepsis

**Fig. 1:**
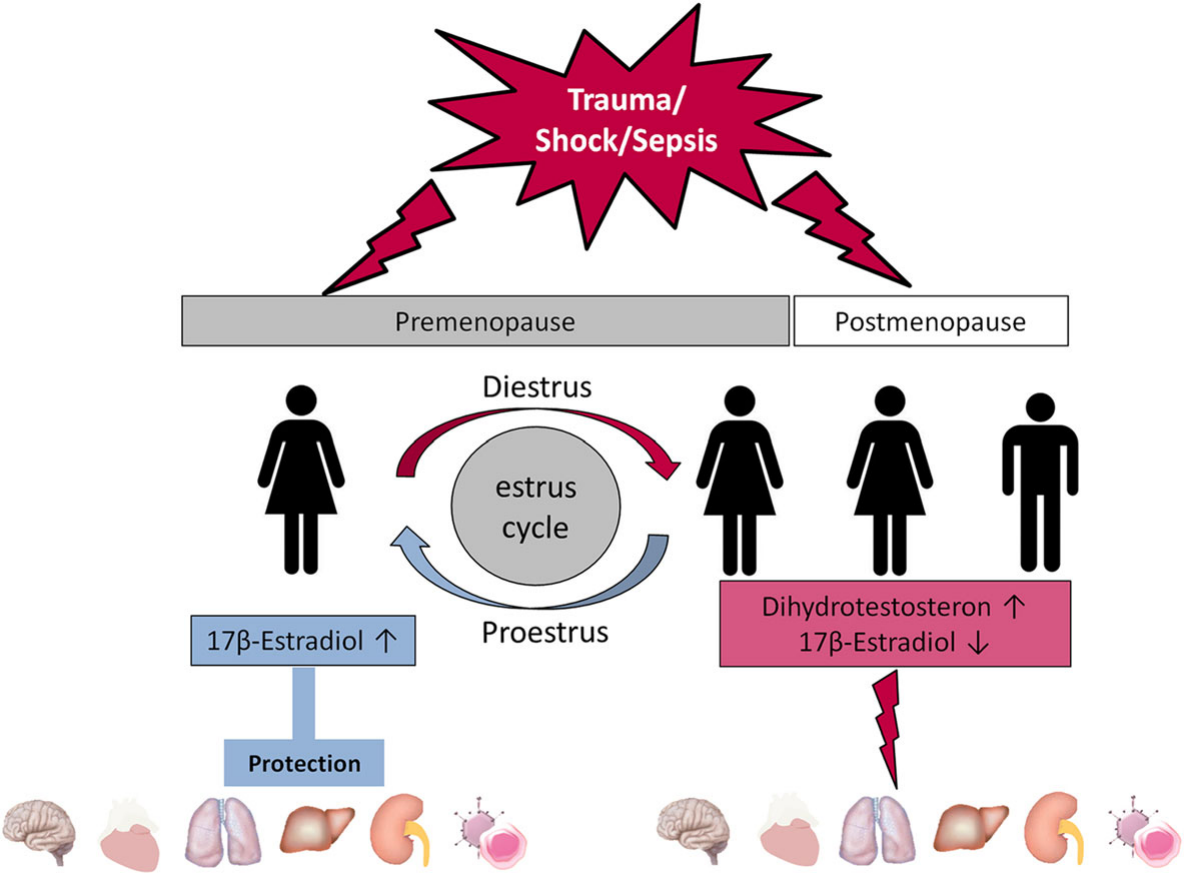
Trauma, shock and sepsis have several deleterious effects on organ systems depending on gender and the prevailing hormonal milieu

As mentioned above, there is evidence for a gender dimorphism in the morbidity and mortality following trauma, hemorrhage and sepsis (Fig. 1). It was reported for the first time in 1975, that males are more prone to posttraumatic infections [34]. Since then, several studies have indicated that male gender and age are major risk factors for infections and multiple organ failure after trauma and blood loss [22, 23, 32–35].

Inflammation represents a common line of defense for maintaining the physiological homeostatic balance following infection or trauma. Subsequently, the inflammatory process leads to complex pro- and anti-inflammatory mechanisms. Additionally, the immune response to acute vs. chronic inflammatory processes is different and must be considered. In clinical reality, those acute and chronic inflammatory processes commonly together occur in the same patient (e.g., a patient with chronic pulmonary obstructive disease and involved in a car accident). This complexity in inflammatory processes, preexisting comorbidities and possible patient medication directly affects the inflammatory response. However, even highly sophisticated animal models cannot reflect this complexity of real life, which may account for other factors in addition to sex hormones, and thus contributes to divergent results between experimental and clinical studies. However, a further discussion of differences in the response to acute vs. chronic inflammatory disease processes is beyond the scope of this review.

The majority of studies also demonstrated an improved outcome in females after trauma. Interestingly, gender itself may not be an independent prognostic factor. Retrospective analyses revealed that female patients had a higher mortality rate if an infection or severe sepsis occurred after trauma [36–38]. In contrast to these findings, other studies reported a significantly better outcome for women after traumatic injury, severe blood loss and sepsis [22, 33, 39]. The contradictory findings are most likely because Eachempati et al. [36] and Napolitano et al. [37] did not consider age and prevailing hormonal milieu as confounders. However, age may play a less important role at the time of injury than hormonal status since hormone blood levels differ significantly during the menstrual cycle. In a large multicenter analysis including more than 20,000 patients, the authors demonstrated a significant survival benefit for female patients younger than 50 years [40]. In accordance with these findings, posttraumatic sepsis and multiple organ failure was reduced in women when age was taken into account [41, 42]. In contrast to the abovementioned studies, clinical findings have demonstrated diminished survival in females following adverse circulatory conditions [43–46]. In this respect, the endocrine milieu in females is regularly influenced by the estrous cycle and by the onset of menopause. In the United States, the average onset of menopause occurs at the age of 50 years [47]. Thus, it is important that age and the prevailing hormonal status be taken into account as a first step in all gender-related studies. Furthermore, exogenous hormones are frequently administered and further influence the hormonal status. The intake of oral contraceptives and hormone replacement therapy is not documented in most clinical studies investigating gender-specific outcomes in critically ill patients. It is estimated that 21% of the women in the United States take hormone replacement therapy, which represents a substantial percentage of female patients [48]. Since no studies were stratified by exogenous hormone treatment or the phase of the estrous cycle, prospective clinical studies in trauma victims that take into account the hormonal status at the time of injury are needed.

### The central nervous system

As mentioned above, sex differences in the immune system and the inflammatory response are evident. Glial cells of the central nervous system are key players in the inflammatory response. These cells mediate the immune response by an inflammatory cytokine burst consisting of tumor necrosis factor α (TNF-α), prostaglandin E_2_ and interleukin-1β (IL-1) [49–51]. The secretion of proinflammatory cytokines is a major step in the deleterious cascade of traumatic brain injury following intra- and extracerebral bleeding, contusion and swelling. This cascade ends in destruction of the blood-brain barrier, reduced cerebral blood flow and necrosis of neuronal cells [52, 53].

There is evidence that after endotoxin injection, female rodents can attenuate systemic inflammation through a reduction of the hypothalamic IL-1 response [54]. This finding is further supported by the fact that the effects of IL-1 administration are estrous cycle-dependent [55]. Moreover, ovariectomy leads to increased IL-1 levels, which can in turn be reduced by administration of estradiol benzoate [56].

Studies have also shown that administration of estrogen 1 hour following traumatic brain injury produces various beneficial effects, such as markedly reduced cerebral edema, decreased neuronal degeneration and improvement of memory and cognitive functions [57–59]. Furthermore, studies have shown that estrogen administration following spinal cord injury also produces salutary effects [60, 61].

### The cardiovascular system

Severe trauma-hemorrhage associated with hemorrhagic shock is a major cause of death [4]. Preservation of cardiac function and vascular responsiveness is crucial for maintaining hemodynamic stability. To achieve stability, fluid management and the use of vasopressors and inotropes are established in intensive care medicine. In this regard, studies have shown that administration of estrogen sulfate following severe blood loss improves outcomes in hemorrhagic shock models [11, 12]. Additionally, following severe blood loss, exogenous estradiol administration exerted protective effects and improved myocardial function, as well as vascular responsiveness [62, 63].

The beneficial effects may be explained by the altered expression levels of heat shock proteins (HSPs) following estrogen administration. The main role of HSPs is to protect cells, and they therefore play an important role in protein folding, apoptosis and signaling [64]. Expression of HSP70 is increased in response to severe blood loss, subsequently leading to a reduced rate of myocardial necrosis [65]. It has been demonstrated that estradiol administration improves cardiac function via upregulation of HSP expression [66–68].

Additionally, Szalay et al. showed that estradiol induces heme oxygenase-1 (HO-1) expression [67]. HO-1 is the rate-limiting enzyme in the degradation of heme into the bioactive signaling molecules free iron, biliverdin and carbon monoxide. In this regard, previous studies have shown that induction of HO-1 and its products exert cardioprotective effects [69, 70].

There is further compelling evidence of a gender dimorphism in the incidence of cardiovascular disease. Males are more prone than females to develop cardiovascular disease and to experience sudden cardiac death [71–73].

### The respiratory system

Patients are highly susceptible to sepsis and multiple organ failure after severe trauma-hemorrhage. Cytokines and adhesion molecules mediate neutrophil infiltration to the lung and subsequent inflammation. These molecules are mainly cytokine-induced neutrophil chemoattractant 1 (CINC-1), CINC-3 and intercellular adhesion molecule 1 (ICAM-1) [74]. Studies have revealed that high levels of female sex hormones attenuated the pulmonary inflammatory response to severe blood loss [75, 76]. Moreover, exogenous estradiol administration mimicked these protective effects in male mice following trauma-hemorrhage. Male animals showed significantly less pulmonary edema and neutrophil infiltration following trauma-hemorrhage and estrogen administration [77]. In accordance with these findings, Doucet et al. demonstrated that ovariectomy had deleterious effects on lung injury following severe blood loss. However, exogenous estradiol administration in those animals could in part improve pulmonary function [78].

The underlying mechanism(s) of the salutary effects of estradiol administration have not been fully elucidated. However, it has been shown that extracellular signal-regulated (ERK) protein kinase partially mediates these effects. Male rats subjected to trauma-hemorrhage showed increased ERK phosphorylation, lung myeloperoxidase activity, and increased levels of IL-6, TNF-α, ICAM-1 and CINC-1, which were attenuated by estradiol administration following trauma-hemorrhage [79].

### The hepatic system

Multiple organ failure subsequent to trauma-hemorrhage, shock and sepsis remains the major cause of death. It is well known that the maintenance of normal hepatic function is pivotal for outcomes following severe traumatic injury [80–82]. Cytokine-mediated tissue inflammation is the first step in the development of sepsis and profound organ damage. Similar to other organ systems, there is also a gender dimorphic response to hepatic injury following trauma-hemorrhage [83–85].

Kupffer cells are hepatic macrophages located in the liver sinusoids and are an important source of proinflammatory chemokines, such as IL-6, IL-10, and TNF-α. It was demonstrated that estradiol treatment downregulated the proinflammatory cytokine burst following trauma-hemorrhage [86, 87]. The salutary effects of post-treatment with estradiol are in part mediated via the p38 mitogen activated protein kinase (MAPK)-dependent HO-1 pathway. Several lines of evidence have established the beneficial effects on hepatic HO-1 induction [88–90]. Severe trauma-hemorrhage resulted in significantly decreased p38 phosphorylation in the liver. Estradiol treatment following trauma-hemorrhage increased p38 phosphorylation and HO-1 induction and attenuated apoptosis. Conversely, administration of a p38 MAPK inhibitor prevented p38 phosphorylation and the increase in HO-1 induction [91].

An additional pathway by which exogenous estradiol exerts its salutary effects following low flow conditions has been shown in further studies. Toll-like receptor 4 (TLR4) is a crucial player in mitochondrial DNA damage and mediates proinflammatory chemokine release [92]. Trauma-hemorrhage led to an increase in TLR4 expression, which was associated with a release of proinflammatory cytokines. However, administration of estradiol following trauma-hemorrhage decreased p38 phosphorylation, as well as levels of the proinflammatory cytokines IL-6, TNF-α, macrophage inflammatory protein-1α (MIP-1α) and MIP-2. Furthermore, estradiol normalized the levels of inducible nitric oxide synthase (iNOS) and adenosine triphosphate (ATP) [92, 93]. In this regard, increased iNOS activity is observed following hepatic tissue injury and is known to be detrimental [94].

According to findings in the cardiovascular system, HSP induction should also be protective following hepatic injury [95]. It was shown that estradiol administration following trauma-hemorrhage induced HSP expression in the injured liver [67, 96, 97]. These findings suggest that the protective effects of estradiol are in part mediated via HSP expression. Furthermore, the reported beneficial effects of estradiol in the hepatic system are mediated via estradiol receptor-α (ER-α) [98]. This was further confirmed by the findings that an ER-α agonist, propyl pyrazole triol (PPT), evoked salutary effects following trauma-hemorrhage. PPT reduced the expression of iNOS, NF-κB and activating protein-1 (AP-1), which are detrimental through their release of proinflammatory chemokines [95]. Moreover, the administration of flutamide, an androgen receptor antagonist, following trauma-hemorrhage prevented hepatic injury in rats subjected to hemorrhagic shock. The salutary effects of flutamide were partially mediated by the estrogen receptor pathways [99]. In addition to ER-α mediated signaling, another estrogen receptor, G protein-coupled receptor 30 (GPR30), has been revealed to play a role in trauma-hemorrhage. GPR30 acts independently of ER and mediates the nongenomic salutary effects of estradiol. Following trauma-hemorrhage, GPR30 acts in a protective manner via the protein kinase A pathway. Alternatively, GPR30 suppression leads to increased apoptosis [100].

### The renal system

Trauma and shock lead to impaired organ function and are associated with a high morbidity and mortality. Acute kidney injury (AKI) is seen in up to 70% of patients with septic shock. Among these patients, the mortality rate reaches nearly 50% [101, 102]. Furthermore, a frequent type of AKI is ischemia-reperfusion injury (IRI). Impaired renal function subsequent to IRI is due to tubular cell damage, apoptosis and the release of proinflammatory cytokines [103–105]. In this respect, studies have revealed gender dimorphism in the susceptibility to AKI. Administration of estradiol attenuated renal IRI whereas testosterone enhanced IRI [106, 107]. Furthermore, administration of estradiol reduced apoptosis and inflammation, and increased endothelial cell survival [108]. Additionally, the levels of proinflammatory TNF-α were reduced and levels of anti-inflammatory IL-10 were increased when estradiol was administered following trauma-hemorrhage. The modulated immune response appears to be due to a decreased number of infiltrating neutrophils [108, 109].

### The immune system

In several disease processes, gender and sex hormones have been shown to affect immunological responses. In this respect, enhanced humoral and cell-mediated immune responses in females are associated with an increased incidence of autoimmune and certain inflammatory diseases (i.e., Hashimoto’s thyroiditis, systemic lupus erythematosus, rheumatoid arthritis, primary biliary cirrhosis and asthma). Further support comes from findings that circulating plasma antibodies are more prevalent in female patients and that women display an increased immune response following immunization [110–116].

The immune response is altered following traumatic injury, and subsequent sepsis, multiple organ failure and mortality is occur more frequently [117–119] in males. Decreased survival rates and a higher frequency of infections and sepsis are reported by large analyses. A registry study of more than 680,000 patients demonstrated a decrease in complication and mortality rates after trauma [120]. A study that included more than 30,000 patients demonstrated that pneumonia is more frequent in males after traumatic injury [33]. Furthermore, in a prospective observational study of 2,183 patients and community-acquired pneumonia, older men had a lower survival rate [121].

Additionally, patients who have undergone surgery are more susceptible to infections. Wichmann et al. found a significant reduction in the number of immune-competent cells in postsurgical men [122]. Moreover, Offner et al. demonstrated gender dimorphism in the onset of postsurgical infections, with male gender as an independent risk factor [123]. The pathogenesis of immune system imbalance is multifactorial. The gender dimorphism is likely due to the divergent expression of pro- and anti-inflammatory cytokines. During sepsis, the secretion of proinflammatory cytokines such as IL-6, IL-8, IL-10, and TNF-α, is increased in male patients [41, 124, 125].

Experimental studies further support these findings. Male mice subjected to polymicrobial sepsis by cecal ligation and puncture showed impaired survival rates compared to female mice [126]. In an experimental endotoxin model, male mice had significantly higher IL-1 blood levels after endotoxin injection [127]. In line with these findings are *in vitro* experiments with human peripheral blood mononuclear cells exposed to endotoxin. The authors demonstrated that proinflammatory TNF-α was significantly higher in endotoxemic male samples; however, administration of estrogen stimulated cytokine expression [128].

It is important to note that it is not the gender but specifically the sex hormones that influence outcome [129]. This is further underscored by the fact that the immune response is more pronounced during the proestrus phase compared to the diestrus phase [56, 130, 131]. Thus, exogenous administration of estrogen enhanced the ER-α-mediated functions of macrophages and dendritic cells [132–134]. Treatment of septic male or ovariectomized female rats with ER-α agonists significantly attenuated sepsis-induced leukocyte-endothelial interactions (rolling, adherent leukocytes and neutrophil extravasation) and improved intestinal integrity [135]. Moreover, following trauma-hemorrhage and subsequent sepsis, administration of estrogen increased the activity of macrophages and survival rates [136].

## Discrepancy of clinical and experimental results

**Fig. 2:**
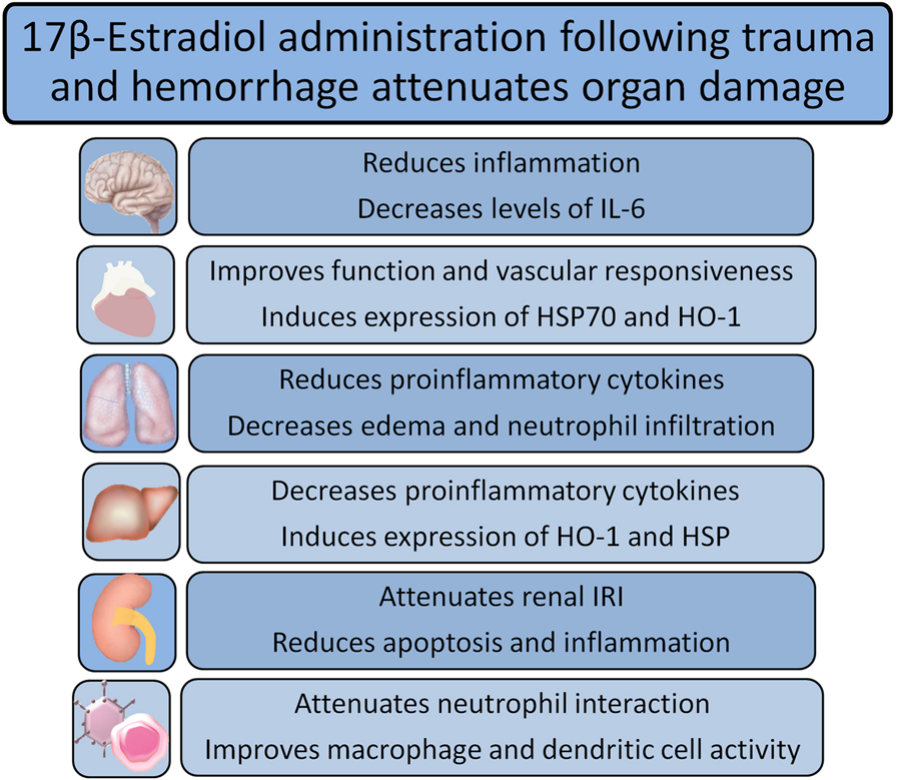
Protective effects of 17β-estradiol on the CNS, heart, lung, liver, kidney and immune cells CNS: central nervous system; HSP: heat shock protein; HO-1: heme oxygenase-1; IRI: ischemia-reperfusion injury; IL-6: interleukin-6

Although the beneficial effects of estrogens on trauma, shock and sepsis have been demonstrated in various studies (Fig. 2), there remains a gap between the bench and the beside. Recently, a nationwide review indicated that female gender represents an independent risk factor for mortality in cases of spontaneous bacterial peritonitis [137]. These findings are in contrast to experimental and clinical results. Although patient number with more than 88,000 is high, those registry-based surveys do have some major limitations. Clinical studies mainly report on heterogeneous populations and are probably hampered by incomplete data sets. Most of these trials lack information regarding hormonal status at the time of injury or the onset of sepsis. Furthermore, information about intake of oral contraceptives, menstrual cycle status and hormone replacement therapy is not provided. Additionally, information should be provided if a female victim is pre- or postmenopausal.

In contrast, experimental studies provide a body of evidence indicating that estrogens are beneficial following adverse circulatory conditions. This might be due in part to the fact that most experimental studies were conducted using young male animals. Moreover, experimental studies follow a highly structured protocol in a homogenous cohort where the use of various agents such as fluid resuscitation (blood, crystalloids or plasma) can be easily defined and controlled, which is usually in contrast to the situations in trauma victims.

## Can estrogens be used to prolong permissive hypotension in the absence of fluid resuscitation?

Frequently, the transportation of the injured from remote areas may be hampered and it may take longer than the “golden hour” for the patient to reach a definitive care center. In light of this, attempts have been made to determine if the interval of permissive hypotension can be increased pharmacologically without fluid resuscitation. Experiments conducted in rats and minipigs showed that administration of estrogens (in a volume of 0.4 ml/kg BW) following major blood loss (60% of the circulating blood volume) maintained permissive hypotension and improved survival rates of animals to over 50% for the examined period of up to 6 hours. Furthermore, if fluid resuscitation was provided at the end of the experiment, it resulted in long-term survival [11, 12, 138, 139]. Thus, administration of estrogens can be carried out at the scene of an accident to stabilize the injured for transportation from rural areas to a definitive care facility for a period involving at least 3 hours. These findings therefore suggest that the so-called “golden hour” can be increased to at least 3 hours for transportation of the injured from the site of injury to definitive care treatment center.

With regards to the mechanism by which EES produces its salutary effects on cardiac functions in the absence of fluid resuscitation, studies have shown this hormone downregulated cardiac NF-κB and restored Nrf2 30 min after EES administration. Furthermore, EES improved but did not restore left ventricular performance at this early interval after treatment. Thus, a major contributor to the beneficial effects of EES on cardiac function following blood loss in the absence of fluid resuscitation is probably via downregulation of cardiac nuclear NF-κB and restoration of cardiac nuclear Nrf2. Furthermore, the restoration of this signaling pathway occurs prior to restoration of cardiac functions [140].

Studies have also shown that major blood loss induces a significant increase in plasma nitrate/nitrite and aortic iNOS. In contrast, trauma-hemorrhage induces a significant decrease in aortic phospho-endothelial NOS (p-eNOS). These alterations correlated closely with trauma-hemorrhage-induced cardiac depression. EES treatment following trauma-hemorrhage downregulated the trauma-hemorrhage-induced increase in plasma nitrate/nitrite and aortic iNOS. Furthermore, it restored p-eNOS expression at 30 min after trauma-hemorrhage-MBO, even in the absence of fluid resuscitation. Thus, the salutary effects of EES on cardiac function following severe blood loss in the absence of fluid resuscitation are linked to the normalization of plasma nitrate/nitrite concentrations, aortic iNOS and restoration of p-eNOS expression [29].

Studies have shown that administration of ICI 182,780 (estrogen receptor antagonist) 30 min prior to EES completely abolished the salutary effect of EES on cardiac function. Furthermore, the specific ER-β antagonist PHTPP, but not the specific ER-α antagonist MPP, completely abrogated the salutary effect of EES on cardiac function at 30 min post-MBO. Thus, the beneficial effects of EES on cardiac function following severe blood loss without fluid resuscitation occur via cardiac estrogen receptors and primarily via cardiac ER-β [141].

Additional studies have shown that trauma-hemorrhage induced a significant decrease in cardiac Bcl-2 and a significant increase in cardiac Caspase-3 and -8. Both signaling alterations were closely correlated with T-H-induced cardiac depression. EES treatment following trauma-hemorrhage without fluid resuscitation restored cardiac Bcl-2 and the trauma-hemorrhage-induced increase in cardiac Caspase-3 and -8. Thus, the major contributing factor to the beneficial effect of EES on cardiac function following severe blood loss appears to be induced via the inhibition of T-H-induced cardiac apoptosis, mediated by restoration of cardiac Bcl-2 and normalization of the T-H-induced increase in cell death signaling pathways [142].

## Conclusion

An abundance of evidence exists highlighting the salutary effects of estrogens following adverse circulatory conditions. Studies reveal that estrogens beneficially influence cytokine release, chemotaxis of neutrophils, expression of HSP, induction of HO-1 and the restoration of organ function following shock and sepsis. Accordingly, estrogens contribute to the higher survival rates in the aforementioned studies. The exact mechanism by which estrogen exerts its beneficial immunomodulatory effects has not been fully elucidated until now. However, there are studies reporting direct and indirect synergistic effects on signaling mechanisms and pathways. Since the hormonal milieu rather than gender influences outcomes after trauma and sepsis, prospective clinical trials are needed to address this issue. It should also be noted that estrogens may be used to prolong the permissive hypotension period and thus aid in the prolonged transportation of the injured from the scene of the accident.

The consideration of gender and sex hormone status for treatment in the clinical arena represents an important and novel step towards personalized medicine.

## Abbreviations

AKI: Acute kidney
AP-1: Activating protein-1
ATP: Adenosine triphosphate
CINC-x: Cytokine-induced neutrophil chemoattractant x
EES: Ethynyl ethinyl estradiol-3sulfate 3 sulfate
ERK: Extracellular signal-regulated protein kinase
ER-α: Estradiol receptor-α
GPR30: G protein-coupled receptor 30
HO-1: Heme oxygenase-1
HSP: Heat shock protein
ICAM-1: Intercellular adhesion molecule-1
IL-x: Interleukin-x
iNOS: Inducible nitric oxide synthase
IRI: Ischemia-reperfusion injury
MAPK: Mitogen activated protein kinase
p-eNOS: Phospho-endothelial nitric oxide synthase
TBI: Traumatic brain injury
TLR4: Toll-like receptor 4
TNF-α: Tumor necrosis factor-α
